# Radiomic signatures associated with longitudinal TNM downstaging for prognostic stratification in breast cancer

**DOI:** 10.1186/s13244-026-02284-7

**Published:** 2026-04-18

**Authors:** Ming Fan, Weihao Liu, Bohan Zhao, Tao Tan, Xunheng Wang, Lihua Li

**Affiliations:** 1 Institute of Biomedical Engineering and Instrumentation, Hangzhou Dianzi University, Xiasha High Education Zone, Hangzhou, China; 2 Faculty of Applied Sciences, Macao Polytechnic University, Macao, China

**Keywords:** Breast cancer, Neoadjuvant chemotherapy, TNM downstaging, Radiomics

## Abstract

**Objectives:**

The tumor-node-metastasis (TNM) staging system is vital for evaluating treatment efficacy in breast cancer patients undergoing neoadjuvant chemotherapy (NACT). However, the prognostic significance of longitudinal TNM changes remains unclear. This study aimed to develop radiomic signatures associated with TNM downstaging (dTNM) and to evaluate their utility in prognostic stratification for breast cancer patients.

**Materials and methods:**

The prognostic analysis included a development cohort (*n* = 292) and two external validation sets (*n* = 180, *n* = 61), all with DCE-MRI data and follow-up information. A random forest-based multitask model was developed using radiomic features from DCE-MRI to predict recurrence, pathological complete response (pCR), and dTNM in the development cohort, stratifying patients into distinct groups. The model’s discriminative performance was assessed with the area under the curve (AUC). In the external validation set, a multivariable Cox proportional hazards model evaluated the prognostic significance of the groups stratified by the radiomic signatures.

**Results:**

The multitask model, incorporating 17 imaging features, achieved AUCs of 0.905, 0.795, and 0.818 for predicting recurrence, pCR, and dTNM, respectively, in the inner validation set from the development dataset. External validation showed that, after adjusting for clinicopathological factors, the dTNM-related radiomic signatures were independently associated with better overall survival (OS) and recurrence-free survival (RFS) (*p* < 0.001 and *p* = 0.004, respectively). Furthermore, group stratification by radiomic signatures associated with pCR and dTNM demonstrated significant differences in survival (all *p* < 0.001) in both external validation datasets.

**Conclusion:**

Radiomic signatures of dTNM can be a prognostic indicator for survival outcomes in breast cancer.

**Critical relevance:**

This study demonstrates that radiomic signatures associated with dTNM offer valuable prognostic insights for survival and recurrence outcomes.

**Key Points:**

- The imaging-based model can predict longitudinal TNM staging.
- Radiomic signatures of dTNM demonstrate significant prognostic value.
- dTNM-associated radiomic signatures provide better prognostic stratification than pCR.

**Graphical Abstract:**

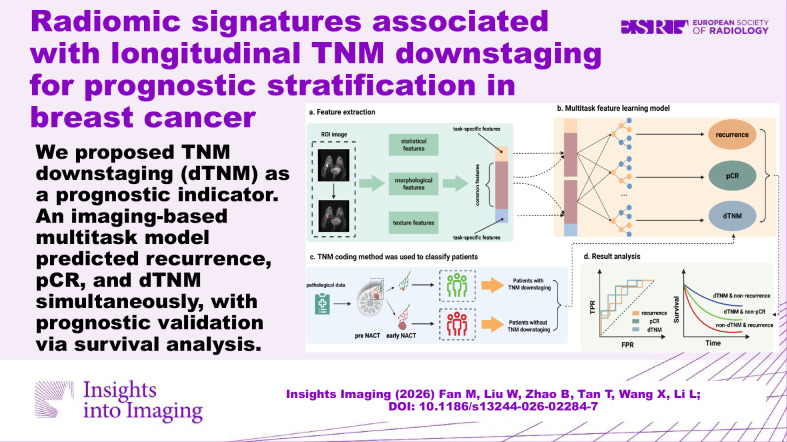

## Introduction

Neoadjuvant chemotherapy (NACT) plays a critical role in breast cancer management by reducing the preoperative tumor size, improving surgical outcomes, lowering recurrence rates, and increasing overall survival (OS) [1, 2]. Pathological complete response (pCR) and residual cancer burden (RCB) are established prognostic biomarkers strongly correlated with favorable long-term survival outcomes [3, 4]. Furthermore, NACT demonstrates systemic efficacy by substantially decreasing the incidence of distant metastases [5]. Early and accurate evaluation of treatment response is essential for guiding therapeutic strategies and facilitating personalized patient management.

Unlike pCR, which provides a binary outcome (presence or absence of residual disease), the tumor-node-metastasis (TNM) staging system offers a more nuanced and quantitative assessment of tumor burden by evaluating tumor size (T), lymph node involvement (N), and metastasis (M) [6]. This stratification not only maps disease progression patterns but also generates prognostic indices for survival prediction. Postneoadjuvant TNM staging further enables serial tumor burden quantification, providing actionable data for treatment escalation or de-escalation decisions and long-term survival modeling [7, 8].

NACT typically involves multiple cycles, making longitudinal analysis of TNM staging crucial for assessing treatment response [9]. TNM downstaging (dTNM), characterized by the regression of tumor characteristics (e.g., T, N, or M categories moving from higher to lower values), serves as a key prognostic marker for favorable outcomes and is essential for longitudinal assessment and treatment optimization [10]. Thus, dTNM is a critical indicator of treatment efficacy [11]. Consequently, the growing recognition of longitudinal time significance in cancer research [12–14] further underscores the value of dTNM analysis for prognostic assessment and treatment optimization.

Dynamic contrast-enhanced magnetic resonance imaging (DCE-MRI) enables noninvasive evaluation of tumor morphological features (e.g., size and shape) and functional properties (e.g., blood flow and vascular permeability), which reflect underlying tumor biology and serve as predictive biomarkers for the NACT response and long-term clinical outcomes [15–17]. Emerging radiomic approaches combine preoperative MRI-derived features (e.g., intratumoral heterogeneity (ITH) indices and radiomic scores) with clinicopathological factors to optimize pCR prediction in breast cancer patients [18]. Furthermore, MRI-driven precision oncology models utilize three-dimensional spatial tumor features to predict progression-free survival (PFS) and OS, thereby enhancing personalized therapeutic strategies [19].

While prior research has employed data-driven approaches incorporating longitudinal imaging features to predict the NACT response, acquiring serial imaging data throughout treatment remains logistically challenging. Although TNM staging is pivotal for breast cancer prognosis, the potential of imaging to predict longitudinal treatment changes preoperatively remains underexplored. This study aimed to analyze DCE-MRI–predicted longitudinal TNM staging and assess its prognostic significance in patients undergoing NACT.

## Materials and methods

### Dataset

The Institutional Review Board (IRB) at Hangzhou Dianzi University (IRB-2019001) approved the study and was exempt from informed consent because of its retrospective design. All the data were obtained from publicly available datasets from The Cancer Imaging Archive (TCIA).

The prognostic development dataset (Duke-Breast-Cancer-MRI) comprised 922 biopsy-confirmed patients with invasive breast cancer who underwent NACT [20]. This dataset contains clinical information, including patient age, race, estrogen receptor (ER) status, progesterone receptor (PR) status, human epidermal growth factor receptor 2 (HER2) status, and TNM staging. The preoperative DCE-MRI was performed prior to biopsy. The M stage prior to NACT was derived from the pathology biopsy report. Through pathological evaluation during the second follow-up, the M stage after NACT was assessed based on the pathological response to NACT. This dataset contains only recurrence data and does not include survival time information.

Two independent datasets were used for prognostic validation: the ISPY1 dataset (*n* = 222) [21] and the NACT-Pilot dataset (*n* = 64) [22], both from the TCIA. Both the ISPY1 and NACT-Pilot datasets include breast cancer patients who underwent NACT, along with preoperative DCE-MRI performed after biopsy, clinical data, and follow-up information. The ISPY1 dataset includes OS and recurrence-free survival (RFS), while the NACT-Pilot dataset includes RFS only.

Follow-up data for both prognostic validation datasets were obtained from electronic medical records. RFS was defined as the time from NACT initiation to local or distant progression, or death from any cause. Follow-up methods were consistent across datasets.

**Fig. 1:**
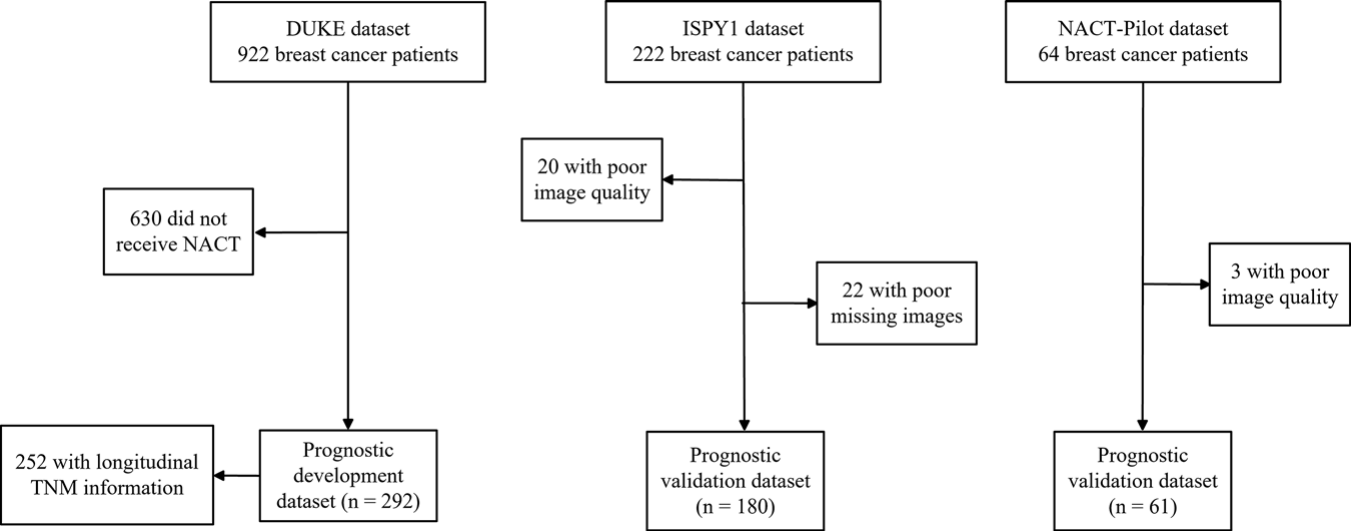
Data overview

The exclusion criteria were as follows: patients with missing or incomplete preoperative DCE-MRI data, those who did not undergo NACT, those with poor-quality images (e.g., with numerous post-biopsy artifacts), and those with incomplete imaging series. Based on the above exclusion criteria, the Duke dataset contains 292 samples and is designated as prognostic development. The ISPY1 dataset, after screening, includes 180 samples, which are labeled as prognostic validation 1. Furthermore, the NACT-Pilot dataset consists of 61 samples, also designated as prognostic validation 2. Detailed data collection for the three datasets is illustrated in Fig. 1.

### Framework overview

**Fig. 2:**
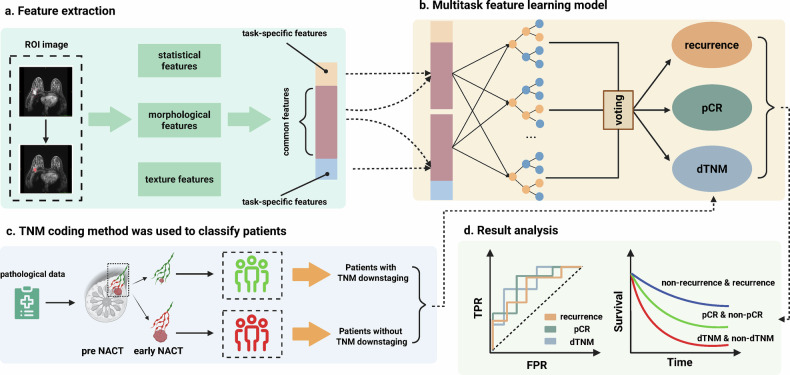
Overview of the framework

The framework of this study is shown in Fig. 2. To investigate longitudinal changes in TNM stage during treatment, we developed a prognostic indicator called dTNM. Radiomics were extracted from the DCE-MRI in the development dataset and used to build the multitask learning model for simultaneously predicting prognostic indicators (recurrence, pCR, and dTNM status).

Our model is designed to predict dTNM solely from pre-treatment MRI, reflecting a clinically relevant scenario where only baseline imaging is available before therapy initiation. In both prognostic validation datasets, the selected imaging features and trained model parameters from the development dataset were applied to predict the three prognostic indicators.

### Prognostic dTNM indicator analysis

During NACT, patients who achieve pCR often demonstrate reductions in tumor (T), lymph node (N), and/or metastasis (M) stages. This study evaluated the significance of dTNM staging during NACT. Patients were classified as experiencing dTNM = 1, if any two of the T, N, and M stages decreased.

**Fig. 3:**
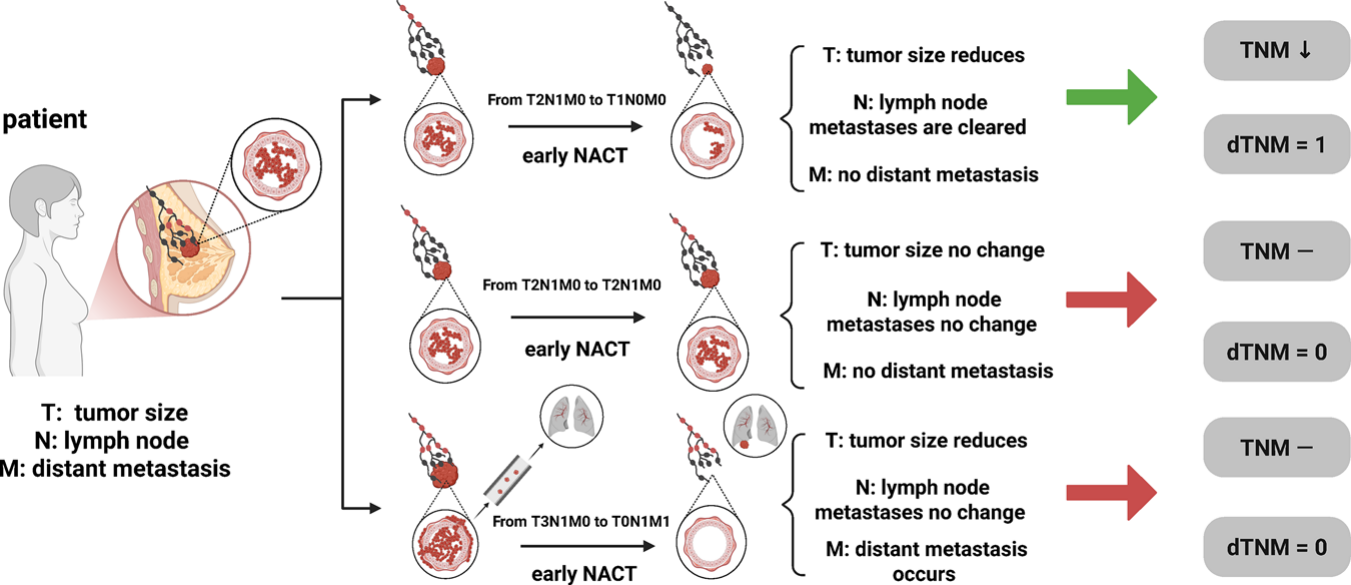
Illustration of the dTNM indicator

Fig. 3 illustrates three specific scenarios of the dTNM definition, including the example of a patient transitioning from T2N1M0 to T1N0M0 and cases where patients show no significant stage changes or disease progression, such as a transition from T3N1M0 to T0N1M1. First, tumor shrinkage (e.g., from T2 to T1), regression of lymph node metastases (e.g., from N1 to N0 confirmed by imaging or pathology), and the absence of distant metastasis (remaining at M0) collectively indicate dTNM = 1. Second, dTNM = 0 is defined by either no changes in T, N, or M stages, or by tumor reduction (e.g., from T3 to T0), unchanged lymph node metastasis (remaining at N1), and the development of distant metastasis (e.g., from M0 to M1). dTNM also reflects changes in clinical staging (e.g., a transition from stage III to stage I corresponds to dTNM = 1) [23]. Supplementary Fig. 1 presents four patient examples of dTNM. It illustrates the TNM staging changes from T3N1M0 to T0N0M0 and from T3N2M0 to T1N0M0, both indicating significant downstaging (Supplementary Fig. 1a, c). Additionally, other examples include TNM staging decreases from T4N1M0 to T1N1M1 and from T2N1M0 to T2N0M0, further illustrating non-dTNM.

### Image preprocessing

The detailed imaging protocols are described in the supplementary Methods. To minimize the effects of varying scanner parameters on image features, DCE-MRI images were standardized by resampling voxels to achieve isotropic dimensions of 0.8 mm. To correct for magnetic field inhomogeneity, the N4 bias field correction algorithm was applied. Intensity normalization was then applied to map the gray values uniformly within the range of 0–800, ensuring consistency for subsequent feature extraction.

For the prognostic development dataset, the tumor annotation boxes were provided and drawn by either 6 or 4 fellowship-trained radiologists. Building on our previous work [12], a semi-automated segmentation approach was employed: the spatial fuzzy C-means (FCM) algorithm was first applied to segment tumor regions of interest (ROIs), followed by manual refinement of tumor boundaries to ensure accuracy. For the prognostic validation datasets 1 and 2, two experienced radiologists, each with over 10 years of expertise, annotated the images by consensus. ROIs were identified from the first post-contrast series (S1), acquired approximately 2 min after contrast injection, when the images display peak enhancement for optimal tumor visualization. Notably, the ROIs included necrotic tumor regions.

Radiomics were extracted from precontrast (S0), postcontrast (S1, S2, and S3), and subtraction images (S1–S0, S2–S0, and S3–S0) by subtracting precontrast from postcontrast images to emphasize tissue changes caused by the contrast agent. Moreover, image subtraction (S2–S1, S3–S1, and S3–S2) was performed to quantify dynamic changes in tissue characteristics during the phases of contrast enhancement, consistent with prior methods [24]. Detailed feature extraction (*n* = 107) is provided in Supplementary Methods.

### Survival analysis

Kaplan‒Meier curves were used to compare survival outcomes, whereas the log-rank test was used to evaluate the significance of any observed differences. Multivariate Cox regression analysis was conducted to determine whether dTNM imaging features were independently associated with survival outcomes after we adjusted for clinicopathological factors, including age, race, ER status, PR status, and human HER2 status. Patients who remained event-free at the end of the 10-year follow-up period were censored at that time point.

### Statistical analysis

To address collinearity among radiomic features, we removed one feature from each highly correlated pair (*r* > 0.9) based on its average correlation with the remaining features. The Chi-square test was used to assess differences in categorical demographic variables between groups.

The prognostic development dataset was divided into training (70%) and internal validation (30%) sets for model evaluation. Model performance was assessed using the area under the curve (AUC) metric on the internal validation set. To identify shared features across tasks, a multitask random forest model was employed [25]. The model learns both task-specific features (relevant to individual tasks) and shared features (relevant to multiple tasks). This is achieved by jointly optimizing the splits for all tasks, allowing the model to capture both the commonalities and differences among them.

The feature importance was determined by the AUC during the node splitting process. Subsequently, recursive feature elimination with cross-validation (RFECV) was used to optimize the feature subset. The hyperparameters were fine-tuned through a grid search in each validation cycle until the optimal model was identified. The top-ranked features from all prediction tasks formed a shared feature subset, enhancing model performance while capturing task-specific correlations. Shapley additive explanations (SHAPs) were employed to analyze each feature’s impact on the model, thereby enhancing the model’s interpretability. A power analysis was conducted to assess the statistical power of the study (Supplementary Methods).

## Results

### Patient characteristics

Table 1 presents the clinical characteristics of patients from the prognostic development dataset (*n* = 292) and two prognostic validation datasets (*n* = 180 and *n* = 61). The clinicopathological variables included age, menopausal status, recurrence, mortality, ER, PR, and HER2 status, treatment response, and TNM stage.

**Table 1:** Patient characteristics

Characteristic	DUKE	ISPY1	NACT-Pilot
Number	292	180	61
Age	48 (21–76)	42.9 (26.7–68.8)	48.1 (29.7–72.4)
Menopausal status			
Premenopausal	166/292 (52.8%)	N/A	N/A
postmenopausal	126/292 (47.2%)	N/A	N/A
Recurrence			
No event	251/292 (85.9%)	131/180 (72.7%)	38/61 (62.3%)
Event	41/292 (14.1%)	49/180 (27.3%)	23/61 (37.7%)
Death			
Event	N/A	33/180 (18.3%)	N/A
No event	N/A	143/180 (79.5%)	N/A
Unknown	N/A	4/180 (2.2%)	N/A
ER			
Positive	169/292 (57.9%)	101/180 (56.1%)	28/61 (45.9%)
Negative	123/292 (42.1%)	77/180 (42.8%)	20/61 (32.8%)
Others	0/292 (0.0%)	2/180 (1.1%)	13/61 (21.3%)
PR			
Positive	135/292 (46.2%)	84/180 (46.7%)	22/61 (36.1%)
Negative	157/292 (53.8%)	94/180 (52.2%)	26/61 (42.6%)
Others	0/292 (0.0%)	2/180 (1.1%)	13/61 (21.3%)
HER2			
Positive	181/292 (62.0%)	52/180 (28.9%)	14/61 (23.0%)
Negative	111/292 (38.0%)	124/180 (68.9%)	31/61 (50.8%)
Others	0/292 (0.0%)	4/180 (2.2%)	16/61 (26.2%)
Pathological response			
PCR	87/292 (29.8%)	45/180 (25.0%)	N/A
Non-PCR	205/292 (70.2%)	135/180 (75.0%)	N/A
TNM status			
Tumor size			
NA	3/292 (1.0%)	N/A	N/A
T1	62/292 (21.2%)	N/A	N/A
T2	159/292 (54.5%)	N/A	N/A
T3	53/292 (18.2%)	N/A	N/A
T4	15/292 (5.1%)	N/A	N/A
Nodes			
NA	3/292 (1.0%)	N/A	N/A
N0	110/292 (37.7%)	N/A	N/A
N1	127/292 (43.5%)	N/A	N/A
N2	31/292 (10.6%)	N/A	N/A
N3	21/292 (7.2%)	N/A	N/A
Metastasis			
MX	63/292 (21.6%)	N/A	N/A
M0	228/292 (78.1%)	N/A	N/A
M1	1/292 (0.3%)	N/A	N/A

The prognostic development dataset consisted of data from women aged 27–74 years, with a mean age of 48 years. Specifically, the prognostic validation 1 dataset included women aged 26.7–68.8 years (mean age 42.9). In this cohort, OS had a median of 3.897 years (range: 0.512–6.761 years), while RFS had a median of 3.778 years (range: 0.490–6.904 years). The prognostic validation 2 dataset included women aged 29.7–72.4 years (mean age 48.1). In this cohort, RFS was reported with a median of 5.720 years (range: 0.276–9.846 years). While OS validation is limited by data availability, RFS provides meaningful complementary evidence for the model’s ability to predict survival outcomes.

### Prognostic analysis of longitudinal TNM staging

We examined the associations between longitudinal TNM status and recurrence (Table 2), revealing that both TNM indicators and changes (dT, dN) from pretreatment to posttreatment were significantly associated with recurrence (*p* < 0.05). Notably, pretreatment nodal status showed the most pronounced association with recurrence (*p* = 0.001). Furthermore, longitudinal TNM changes (dTNM) exhibited even stronger associations with recurrence (*p* < 0.001), and this indicator was associated with pCR (*p* = 0.0219). These findings suggest that patients who achieve dTNM are more likely to achieve pCR and have a favorable prognosis.

**Table 2:** Associations between TNM indicators and recurrence

TNM indicators^*^	*p* value	TNM indicators^†^	*p* value	TNM indicators^‡^	*p* value
T	0.026	T	0.015	dT	0.014
N	0.001	N	0.026	dN	0.014
M	0.134	M	0.305	dM	0.091
-	-	-	-	dTNM	< 0.001

^*^ Preoperative TNM status

^†^ TNM status in the early NACT stage

^‡^ Longitudinal TNM status. The *p* value was calculated using the χ^2^ test

### Multitask learning model for predicting prognostic indicators

We established a model that utilized clinical features, including age, race, ER, PR, and HER2, as a baseline to predict recurrence, pCR, and dTNM indicators. This model achieved AUCs of 0.602, 0.583, and 0.557, respectively (Table 3).

**Table 3:** Radiomics-based single-task and multitask models, along with clinical-based models for predicting prognostic indicators

	AUC (95% CI)	Accuracy (95% CI)	Specificity (95% CI)	Sensitivity (95% CI)
Recurrence^*^	0.602 (0.441–0.766)	0.557 (0.453–0.656)	0.526 (0.416–0.635)	0.750 (0.468–0.911)
pCR^*^	0.583 (0.448–0.715)	0.602 (0.498–0.698)	0.581 (0.457–0.695)	0.654 (0.462–0.806)
dTNM^*^	0.557 (0.426–0.693)	0.605 (0.493–0.708)	0.632 (0.473–0.766)	0.579 (0.422–0.721)
Recurrence^†^	0.829 (0.697–0.925)	0.830 (0.738–0.894)	0.823 (0.724–0.891)	0.889 (0.565–0.980)
pCR^†^	0.701 (0.572–0.811)	0.659 (0.555–0.750)	0.667 (0.541–0.773)	0.643 (0.458–0.793)
dTNM^†^	0.705 (0.580–0.820)	0.724 (0.614–0.812)	0.647 (0.479–0.785)	0.786 (0.641–0.883)
Recurrence^‡^	0.905 (0.790–0.977)	0.898 (0.817–0.945)	0.899 (0.813–0.948)	0.889 (0.565–0.980)
pCR^‡^	0.795 (0.672–0.908)	0.773 (0.675–0.848)	0.768 (0.656–0.852)	0.789 (0.567–0.915)
dTNM^‡^	0.818 (0.712–0.913)	0.803 (0.700–0.877)	0.821 (0.644–0.921)	0.792 (0.657–0.883)

^*^ Clinical-based model

^†^ Single-task model

^‡^ Multitask model

**Fig. 4:**
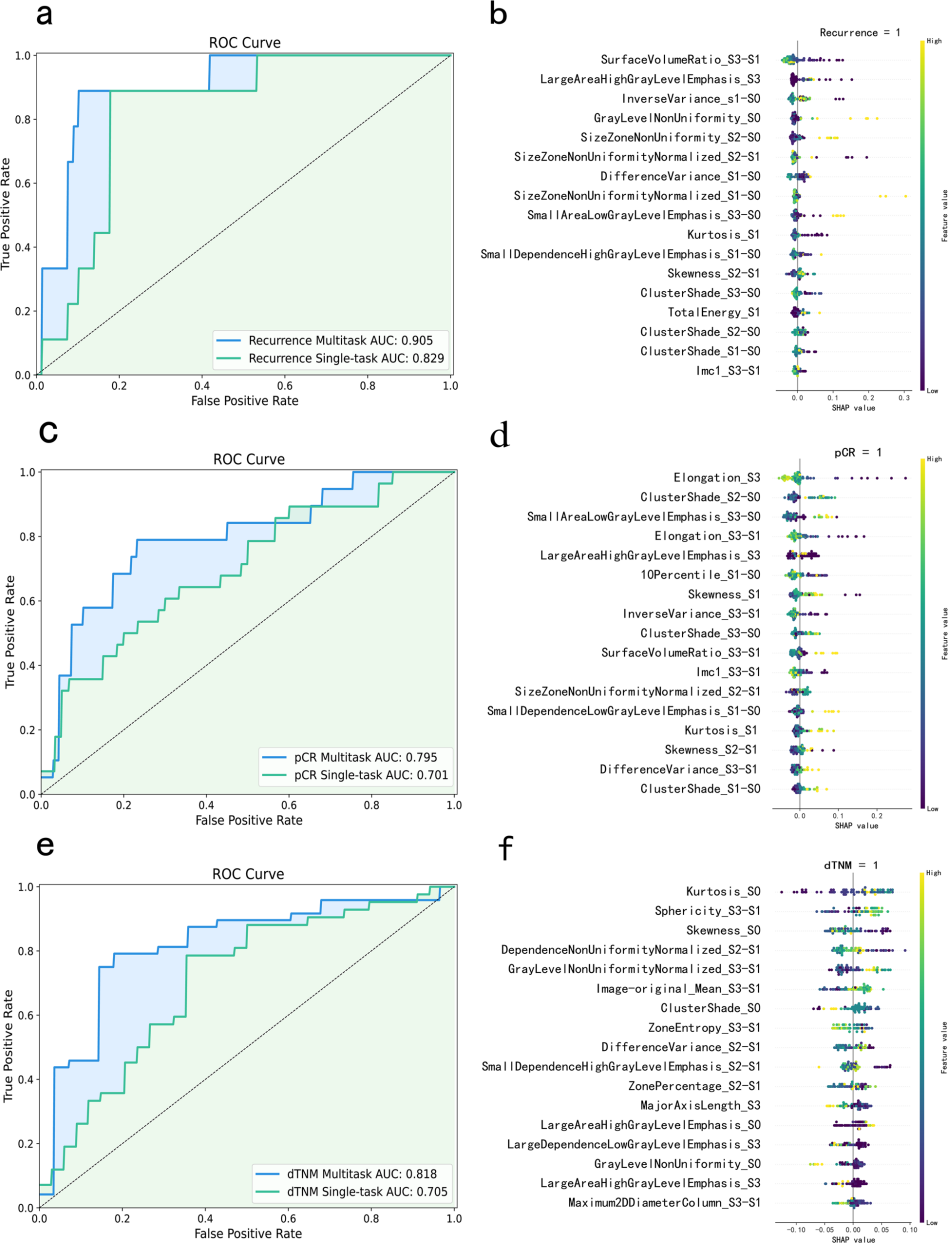
Internal validation of imaging feature-based prognostic indicators: **a** ROC curves comparing multitask and single-task models for recurrence prediction (AUC: 0.905 vs 0.829), with corresponding (**b**) SHAP bee swarm plot for the multitask model’s recurrence prediction; **c** ROC curves comparing multitask and single-task models for pCR prediction (AUC: 0.795 vs 0.701), with corresponding (**d**) SHAP bee swarm plot for the multitask model’s pCR prediction; **e** ROC curves comparing multitask and single-task models for dTNM prediction (AUC: 0.818 vs 0.705), with corresponding (**f**) SHAP bee swarm plot for the multitask model

Additionally, we evaluated a radiomics-based model for predicting these prognostic indicators, utilizing both single-task and multitask learning frameworks. For internal validation, the radiomics-based multitask model achieved an AUC of 0.905 for recurrence, 0.795 for pCR, and 0.818 for dTNM, significantly outperforming both the baseline model and the single-task model (Fig. 4a, c, and e). After integrating clinical and radiomic features, the model demonstrated enhanced predictive performance for recurrence, pCR, and dTNM, with AUC values of 0.916, 0.806, and 0.829, respectively (Supplementary Table 1).

We calculate the SHAP values for each feature across all the samples in the multitask learning model. For both recurrence and pCR prediction tasks, most feature SHAP values were clustered in the negative region. This suggests that the model primarily learned features associated with non-recurrence and non-pCR outcomes. This finding aligns with the distribution patterns observed in the development dataset.

Notably, shared features between recurrence and pCR predictions (e.g., inverse variance and large area high gray level emphasis) significantly contributed to the model’s performance. Similarly, the shared feature between recurrence and dTNM stages (difference variance) also played a key role.‌

These results underscore the advantage of multitask-based radiomic features in enhancing predictive performance and classification accuracy across all three predictive tasks, compared to clinical variables alone or single-task models.

### External validation of the radiomic signatures for survival stratification

The prognostic implications of imaging-derived indicators developed on the development dataset were externally validated in independent validation datasets. In the prognostic validation 1 dataset, the predicted recurrence rate was strongly positively correlated with both OS and RFS (all *p* < 0.001) (Supplementary Figs. 2a, b). However, the predicted pCR was not significantly correlated with OS (*p* = 0.158) but was positively associated with RFS (*p* = 0.008) (Supplementary Fig. 2c, d). Additionally, the dTNM-associated radiomic signature was strongly positively correlated with better OS (*p* < 0.001) and RFS (*p* = 0.004) (Supplementary Fig. 2e, f), and outperformed the pCR indicator. For the prognostic validation 2 dataset, the imaging model-derived recurrence, pCR, and dTNM features were strongly positively correlated with RFS (all *p* < 0.001) (Supplementary Fig. 3a, c, and e, respectively).

We performed subgroup analyses based on tumor subtypes: hormone receptor (HR)-positive (ER and/or PR positive), triple-negative, and HER2-overexpressing (ER negative, PR negative, HER2 positive) (Supplementary Fig. 4). In the prognostic validation 1 dataset, the dTNM-associated radiomic signature showed significant correlations with OS and RFS (both *p* < 0.05). No significant association was observed in the prognostic validation 2 dataset, likely due to the small sample size within subgroups (e.g., *n* = 25 in the HR-positive group).

### External validation of imaging-predicted prognostic indicators in subgroup analysis

**Fig. 5:**
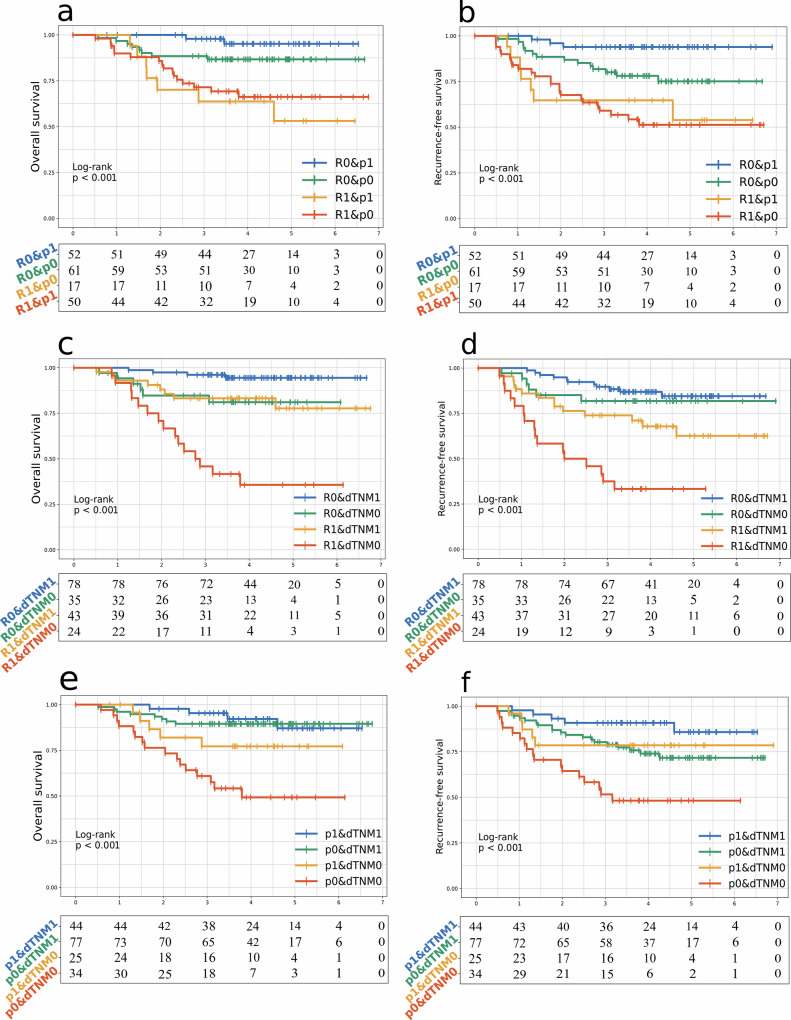
External survival validation of longitudinal TNM indicators and subgroup analysis. The imaging-based model-predicted recurrence and pCR predictions were employed for validating (**a**) OS and (**b**) RFS. Similarly, imaging-based model-predicted recurrence and radiomic signature of dTNM were applied to assess (**c**) OS and (**d**) RFS. Additionally, imaging-based signatures of pCR and dTNM were utilized to evaluate (**e**) OS and (**f**) RFS. R0 denotes recurrence = 0, R1 represents recurrence = 1; p0 indicates pCR = 0, p1 signifies pCR = 1; dTNM0 corresponds to dTNM = 0, while dTNM1 stands for dTNM = 1. dTNM is used in the figure to maintain formatting consistency with recurrence and pCR, but it essentially refers to the radiomic signature of dTNM

In the prognostic validation 1 dataset, patients were stratified based on imaging model-derived recurrence, pCR, and dTNM, and their prognostic significance was evaluated. Patients predicted to achieve pCR without recurrence had significantly better survival outcomes than those not predicted to achieve pCR with recurrence (*p* < 0.001 for both OS and RFS) (Fig. 5a, b). Similarly, patients predicted to have recurrence with non-dTNM had worse survival outcomes than those without recurrence but with dTNM (*p* < 0.001 for both OS and RFS) (Fig. 5c, d).

Furthermore, stratification by radiomic signature associated with pCR and dTNM demonstrated significant differences in survivals (*p* < 0.001 for both OS and RFS) (Fig. 5e, f). Patients classified as non-pCR and non-dTNM had the poorest survival outcomes. OS was similar in the dTNM group irrespective of pCR status; however, the non-dTNM group exhibited lower median survival, and among them, non-pCR patients fared the worst. These findings highlight that, in the prognostic validation 1 dataset, incorporating the imaging model-derived dTNM improves the precision of patient stratification for both OS and RFS.

In the prognostic validation 2 dataset, which contains only RFS data, stratification based on predicted recurrence and pCR demonstrated significant differences in RFS (*p* < 0.001) (Supplementary Fig. 3b). Patients predicted to have recurrence and unlikely to achieve pCR exhibited the poorest survival outcomes. Similarly, stratification by radiomic signature associated with recurrence and dTNM revealed significant differences in RFS (*p* < 0.001) (Supplementary Fig. 3d). Patients classified as non-dTNM exhibited lower survival rates, particularly those in the recurrence and non-dTNM group. Patients with non-dTNM had poorer survival rates, especially those in the non-pCR and non-dTNM group (*p* < 0.001) (Supplementary Fig. 3f). The results are consistent with the findings from the prognostic validation 1 dataset.

### Multivariate survival validation of imaging-based dTNM indicators

In the prognostic validation 1 dataset, the prognostic value of the predicted imaging indicators was evaluated (Table 4 and Supplementary Fig. 5). Recurrence was excluded from this analysis because it is a well-established prognostic factor. The results showed that after clinicopathological variables (i.e., age, race, ER status, PR status, and HER2 status) were adjusted, imaging-derived dTNM status was significantly associated with both OS (HR = 0.230; 95% CI = 0.110–0.486; *p* < 0.001) and RFS (HR = 0.416; 95% CI = 0.228–0.758; *p* = 0.004). A multivariate Cox regression analysis incorporating RCB indicated that imaging-derived dTNM status remained independently associated with both OS (HR = 0.243; 95% CI: 0.113–0.525; *p* < 0.001) and RFS (HR = 0.454; 95% CI: 0.238–0.867; *p* = 0.017) (Supplementary Fig. 6).

**Table 4:** Multivariate analysis of the radiomic signatures and clinicopathological factors

	OS		RFS	
Feature	HR (95% CI)	*p* value	HR (95% CI)	*p* value
Age	0.993 (0.954–1.034)	0.743	0.967 (0.935–1.000)	0.052
Race	1.029 (0.732–1.446)	0.869	0.870 (0.632–1.198)	0.394
ER	0.185 (0.060–0.573)	0.003	0.286 (0.118–0.694)	0.006
PR	1.302 (0.419–4.049)	0.649	1.198 (0.486–2.955)	0.695
HER2	1.282 (0.613–2.680)	0.510	1.327 (0.712–2.473)	0.374
Predicted dTNM	0.230 (0.110–0.486)	< 0.001	0.416 (0.228–0.758)	0.004
Predicted pCR	0.408 (0.182–0.911)	0.029	0.288 (0.140–0.594)	< 0.001

Predicted dTNM refers to the radiomic signature associated with dTNM

In the prognostic validation 2 dataset, imaging model-predicted dTNM status was significantly associated with RFS (HR = 0.137; 95% CI = 0.022–0.879; *p* = 0.034) (Supplementary Fig. 7). As an independent prognostic factor, this radiomic signature was a stronger predictor than clinicopathological indicators (e.g., HER2, PR), underscoring its clinical value in breast cancer prognosis.

### Analysis of radiomic features associated with prognostic indicators

Supplementary Fig. 8 shows the four imaging features that significantly contributed to the imaging model associated with pCR in the prognostic validation 1 dataset, including inverse variance, 10 percentiles, elongation, and cluster shade. Additionally, six imaging features that significantly contributed to the imaging model associated with dTNM are shown, including sphericity, difference variance, gray level nonuniformity, small dependence, high gray level emphasis, and cluster shade. In the prognostic validation 1 dataset, the results revealed that the pCR-associated inverse variance feature was significantly correlated with OS and RFS (Supplementary Fig. 8a, c), and the dTNM-associated gray level nonuniformity feature was also significantly associated with patient survival outcomes (Supplementary Fig. 8b, d). These radiomic features have been reported as imaging biomarkers of ITH [26, 27].

### Case study of prognostic indicator-associated imaging features

**Fig. 6:**
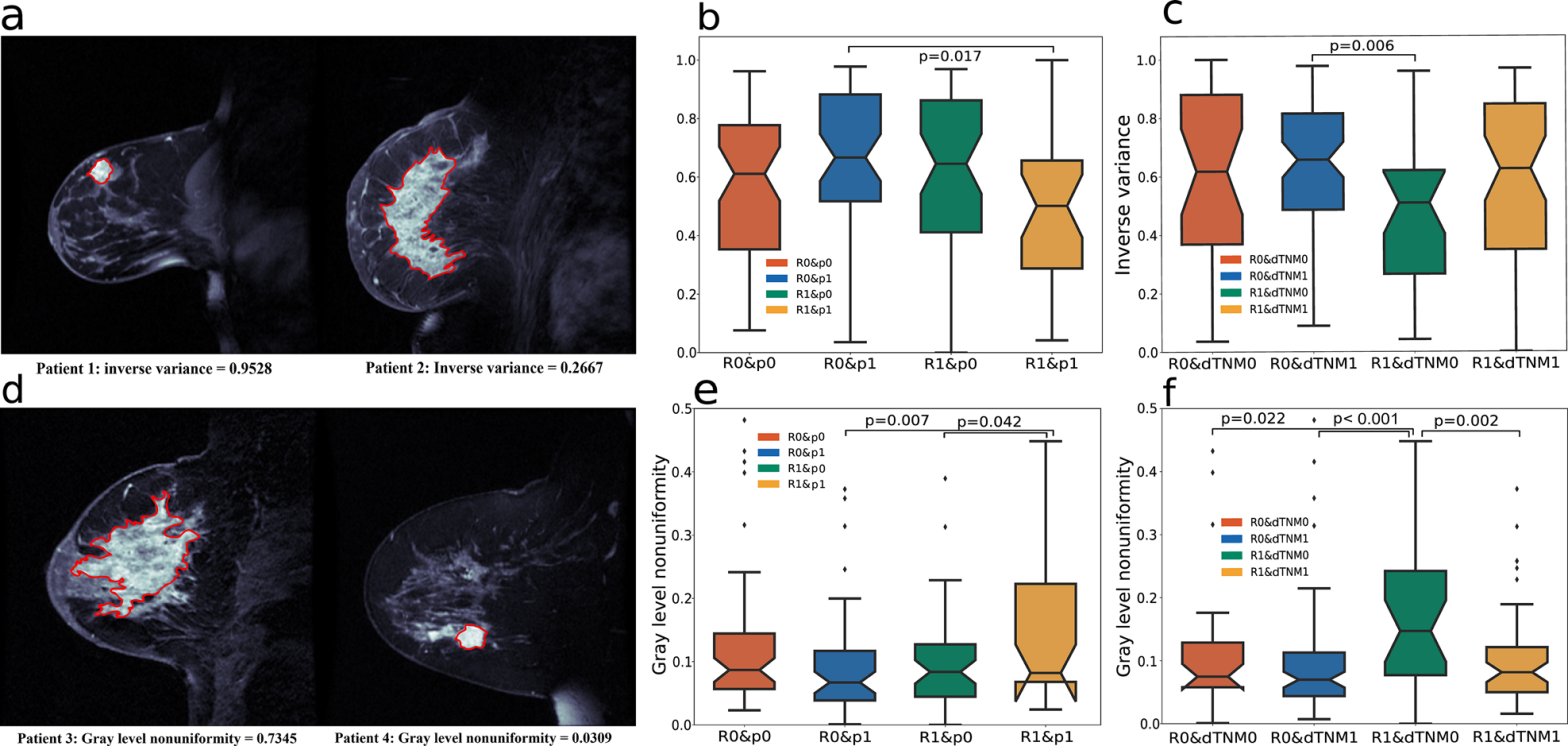
Case study of imaging features, including inverse variance and gray level nonuniformity‌. The lesion areas are outlined in red. **a** The left panel shows a patient (aged 47.02 years) from the predicted R0 & p1 or R0 & dTNM1 group; the right panel shows a patient (aged 49.10 years) from the predicted R1 & p1 or R1 & dTNM0 group. **b** Distribution of inverse variance across stratified groups by predicted recurrence and pCR. **c** Groups stratified by predicted recurrence and dTNM. **d** The left panel shows a patient (age 63.63 years) from the predicted R0 & p1 or R0 & dTNM1 group; the right panel shows a patient (age 52.45 years) from the predicted R1 & p1 or R1 & dTNM0 group. **e** Gray-level nonuniformity feature distribution across groups stratified by recurrence and pCR. **f** Groups stratified by recurrence status and dTNM. ‌Note‌: R0 & dTNM1 indicate no recurrence (recurrence = 0) with, dTNM = 1, whereas R0 & p1 represent no recurrence (recurrence = 0) with pCR (pCR = 1). The other subgroups followed analogous criteria.

Examples of feature values in patients with different prognoses from the prognostic validation 1 dataset are shown in Fig. 6. Patients predicted as non-recurrence with pCR had higher inverse variance values than those predicted as recurrence with pCR. Similarly, patients with model-inferred prognostic indicators of non-recurrence and dTNM had higher inverse variance values than those predicted as recurrence and non-dTNM. Moreover, patients whose model-inferred prognostic indicators were recurrence and non-dTNM had the lowest inverse variance compared with the other patients. This feature measures the homogeneity of gray level variations within an image, where higher values indicate smoother textures and lower heterogeneities.

Notably, gray-level nonuniformity was significantly greater in patients with pCR and recurrence, as well as in those with recurrence and non-dTNM (Fig. 6f). This texture feature measures the variability of gray-level intensity, with higher values indicating greater heterogeneity due to irregular gray level distributions. These findings suggest that the dTNM is more effective than the pCR for identifying imaging features related to prognostic information.

## Discussion

In this study, we developed a novel prognostic indicator based on quantitative longitudinal TNM assessment and used imaging as a surrogate to evaluate its efficacy in predicting breast cancer survival. The proposed indicator specifically reflects whether patients achieve dTNM during NACT. Our findings demonstrate that the dTNM-associated radiomic signature is an independent predictor of OS in the prognostic validation 1 dataset and of RFS across both prognostic validation datasets, supporting its potential utility for breast cancer prognosis.

Previous studies have shown that changes in tumor size, lymph node metastasis status, and tumor markers before and after the initiation of NACT can serve as effective predictors of the final treatment response [28]. Based on this, our model leverages preoperative DCE-MRI to predict the longitudinal changes of TNM staging during NACT. This enhances the clinical utility of the prognostic radiomic signatures complementary to RCB, supporting real-time tumor response monitoring and facilitating timely, personalized interventions.

Previous studies have demonstrated an association between TNM staging and both pCR and recurrence [29, 30]. Similarly, our radiomic signatures associated with dTNM significantly correlated with both outcomes. Specifically, patients predicted as dTNM were more likely to achieve pCR. This is because patients who experience dTNM typically exhibit better responses to NACT, resulting in faster tumor shrinkage and reduced lymph nodes, thereby increasing the likelihood of achieving pCR [31]. They also show lower recurrence risk and improved survival, likely due to decreased tumor burden, enhancing treatment effectiveness [32]. Moreover, dTNM adds prognostic value beyond pCR and RCB; for example, some patients in the Duke breast MRI dataset achieved pCR but without dTNM, yet experienced recurrences. Our analysis confirms that imaging model-predicted dTNM is an independent prognostic factor after adjusting for RCB and clinicopathological variables.

In the external prognostic validation, patients who were predicted as dTNM had survival outcomes superior to those of patients who achieved pCR. In subgroup analysis for external validation of indicators, among patients predicted to achieve pCR, those with predicted dTNM demonstrated significantly better survival outcomes compared to those without downstaging (non-dTNM). The highest survival rates were observed in patients predicted to have dTNM and a recurrence-free status. Among high-risk patients (predicted recurrence or failure to achieve pCR), predicted dTNM was significantly associated with better survival outcomes. Incorporating imaging-derived dTNM into traditional prognostic indicators, such as pCR, enables more detailed patient stratification and highlights the enhanced prognostic value of TNM staging.

We analyzed the important contributions of imaging features, including inverse variance and gray level nonuniformity, in the prognostic evaluation of breast cancer patients based on predicted pCR and dTNM. Specifically, higher inverse variance values indicate greater texture heterogeneity in the image and a greater degree of tumor heterogeneity, suggesting a poorer treatment response. Therefore, it is typically more difficult to achieve pCR in tumors with higher inverse variance values [33]. Higher gray-level nonuniformity usually indicates a more complex internal tissue structure, resulting in a poorer treatment response. This is consistent with the previous study reporting that tumors exhibiting more uniform gray levels tend to shrink significantly during treatment, resulting in dTNM [34].

This study has several limitations. First, although the prognostic value of the dTNM-associated radiomic signatures was evaluated, the absence of TNM staging information in the external validation dataset prevents direct validation of the model, thereby limiting the assessment of its external applicability. Second, although we integrated multiple imaging data and clinical indicators, the relatively small sample size may limit the generalizability of the model, potentially affecting its accuracy and applicability across diverse populations. Third, variability in scanners and acquisition parameters across centers results in inconsistent image quality, which compromises the consistency of radiomic feature extraction and undermines the robustness and reproducibility of predictive models. This limitation highlights the need for larger, more diverse datasets and, importantly, the implementation of internationally standardized imaging protocols. Fourth, the public datasets used in this study lack information on biopsy marker clips for accurate postoperative tumor bed localization. This absence may cause errors in tumor bed localization and consequently introduce uncertainty in pCR determination and post-treatment TNM staging labels. Such label noise could impair the accuracy and generalizability of imaging-based prediction models, particularly for longitudinal TNM evaluation.

In this study, we developed dTNM-associated radiomic features and evaluated their prognostic implications in breast cancer. Integrating imaging-derived dTNM status with established prognostic indicators such as pCR facilitates more accurate patient stratification. In clinical practice, as a noninvasive, imaging-based tool, the prognostic radiomic signatures can complement traditional pathological assessments by providing additional risk stratification. Consequently, the proposed radiomic signature holds promise as a biomarker that facilitates individualized treatment planning. Taken together, the proposed longitudinal prognostic imaging model offers an alternative approach to assessing breast cancer outcomes and personalizing treatment strategies.

## Supplementary information

ELECTRONIC SUPPLEMENTARY MATERIAL

## Data Availability

The prognostic development dataset (DUKE) and two prognostic validation datasets (ISPY1 and NACT-Pilot) are publicly accessible through TCIA and are available at (https://www.cancerimagingarchive.net/collection/duke-breast-cancer-mri/), (https://www.cancerimagingarchive.net/collection/ispy1/), and (https://www.cancerimagingarchive.net/collection/breast-mri-nact-pilot/), respectively.

## Abbreviations

AUC: Area under the curve
DCE-MRI: Dynamic contrast-enhanced magnetic resonance imaging
dTNM: TNM downstaging
ER: Estrogen receptor
HER2: Human epidermal growth factor receptor 2
NACT: Neoadjuvant chemotherapy
OS: Overall survival
pCR: Pathological complete response
PR: Progesterone receptor
RCB: Residual cancer burden
RFS: Recurrence-free survival
ROI: Regions of interest
SHAP: Shapley additive explanations
TCIA: The cancer imaging archive
TNM: Tumor node metastasis

## References

[1] Klein J, Tran W, Watkins E et al (2019) Locally advanced breast cancer treated with neoadjuvant chemotherapy and adjuvant radiotherapy: a retrospective cohort analysis. BMC Cancer 19:306 30943923 10.1186/s12885-019-5499-2 PMC6448234

[2] Spring LM, Bar Y, Isakoff SJ (2022) The evolving role of neoadjuvant therapy for operable breast cancer. J Natl Compr Cancer Netw 20:723–734 10.6004/jnccn.2022.7016 35714678

[3] Spring LM, Fell G, Arfe A et al (2020) Pathologic complete response after neoadjuvant chemotherapy and impact on breast cancer recurrence and survival: a comprehensive meta-analysis. Clin Cancer Res 26:2838–2848 32046998 10.1158/1078-0432.CCR-19-3492 PMC7299787

[4] von Minckwitz G, Untch M, Blohmer JU et al (2012) Definition and impact of pathologic complete response on prognosis after neoadjuvant chemotherapy in various intrinsic breast cancer subtypes. J Clin Oncol 30:1796–1804 22508812 10.1200/JCO.2011.38.8595

[5] Maur M, Guarneri V, Frassoldati A, Conte PF (2006) Primary systemic therapy in operable breast cancer: clinical data and biological fall-out. Ann Oncol 17:v158–v164 16807447 10.1093/annonc/mdj973

[6] Garcia-Tejedor A, Fernandez-Gonzalez S, Ortega R et al (2021) Can we avoid axillary lymph node dissection in N2 breast cancer patients with chemo-sensitive tumours such as HER2 and TNBC? Breast Cancer Res Treat 185:657–666 33068198 10.1007/s10549-020-05970-2

[7] Bertero L, Massa F, Metovic J et al (2018) Eighth edition of the UICC classification of malignant tumours: An overview of the changes in the pathological TNM classification criteria-What has changed and why? Virchows Arch 472:519–531 29209757 10.1007/s00428-017-2276-y

[8] Chen Y, Qi Y, Wang K (2023) Neoadjuvant chemotherapy for breast cancer: an evaluation of its efficacy and research progress. Front Oncol 13:1169010 37854685 10.3389/fonc.2023.1169010 PMC10579937

[9] Ji H, Hu C, Yang X et al (2023) Lymph node metastasis in cancer progression: molecular mechanisms, clinical significance and therapeutic interventions. Signal Transduct Target Ther 8:367 37752146 10.1038/s41392-023-01576-4 PMC10522642

[10] Curigliano G, Burstein H, Gnant M et al (2023) Understanding breast cancer complexity to improve patient outcomes: the St Gallen international consensus conference for the primary therapy of individuals with early breast cancer 2023. Ann Oncol 34:970–986 37683978 10.1016/j.annonc.2023.08.017

[11] Furrer MA, Papa N, Luetolf S et al (2022) A longitudinal study evaluating interim assessment of neoadjuvant chemotherapy for bladder cancer. BJU Int 130:306–313 34418255 10.1111/bju.15579

[12] Fan M, Wang K, Pan D et al (2024) Radiomic analysis reveals diverse prognostic and molecular insights into the response of breast cancer to neoadjuvant chemotherapy: a multicohort study. J Transl Med 22:637 38978099 10.1186/s12967-024-05487-y PMC11232151

[13] Thommen L, Heidrich V, de Andrade RV et al (2024) Abstract PO4-03-04: characterization of gut microbiome of patients with early-stage breast cancer treated with neoadjuvant chemotherapy. Cancer Res 84:PO4-03-04–PO04-03-04

[14] Gao Y, Ventura-Diaz S, Wang X et al (2024) An explainable longitudinal multi-modal fusion model for predicting neoadjuvant therapy response in women with breast cancer. Nat Commun 15:9613 39511143 10.1038/s41467-024-53450-8 PMC11544255

[15] Huang Y, Cao Y, Hu X et al (2023) Early identification of pathologic complete response to neoadjuvant chemotherapy using multiphase DCE-MRI by Siamese network in breast cancer: a longitudinal multicenter study. J Magn Reson Imaging. 10.1002/jmri.29188 10.1002/jmri.29188 38109316

[16] Ogier du Terrail J, Leopold A, Joly C et al (2023) Federated learning for predicting histological response to neoadjuvant chemotherapy in triple-negative breast cancer. Nat Med 29:135–146 36658418 10.1038/s41591-022-02155-w

[17] Jiang Y, Zhang Z, Wang W et al (2023) Biology-guided deep learning predicts prognosis and cancer immunotherapy response. Nat Commun 14:5135 37612313 10.1038/s41467-023-40890-x PMC10447467

[18] Shi Z, Huang X, Cheng Z et al (2023) MRI-based quantification of intratumoral heterogeneity for predicting treatment response to neoadjuvant chemotherapy in breast cancer. Radiology 308:e222830 37432083 10.1148/radiol.222830

[19] Paverd H, Zormpas-Petridis K, Clayton H, Burge S, Crispin-Ortuzar M (2024) Radiology and multi-scale data integration for precision oncology. NPJ Precis Oncol 8:158 39060351 10.1038/s41698-024-00656-0 PMC11282284

[20] Saha A, Harowicz MR, Grimm LJ et al (2018) A machine learning approach to radiogenomics of breast cancer: a study of 922 subjects and 529 DCE-MRI features. Br J Cancer 119:508–516 30033447 10.1038/s41416-018-0185-8 PMC6134102

[21] Hylton NM, Gatsonis CA, Rosen MA et al (2016) Neoadjuvant chemotherapy for breast cancer: functional tumor volume by MR imaging predicts recurrence-free survival-results from the ACRIN 6657/CALGB 150007 I-SPY 1 TRIAL. Radiology 279:44–55 26624971 10.1148/radiol.2015150013 PMC4819899

[22] Dempsey P (2006) 1–13 MRI measurements of breast tumor volume predict response to neoadjuvant chemotherapy and recurrence-free survival. Breast Dis Year Book Q 17:46–47 10.2214/ajr.184.6.01841774 15908529

[23] Park Y, Lee S, Cho E et al (2011) Clinical relevance of TNM staging system according to breast cancer subtypes. Ann Oncol 22:1554–1560 21242587 10.1093/annonc/mdq617

[24] Fan M, Cui Y, You C et al (2022) Radiogenomic signatures of oncotype DX recurrence score enable prediction of survival in estrogen receptor-positive breast cancer: a multicohort study. Radiology 302:516–524 34846204 10.1148/radiol.2021210738

[25] Fan M, Yuan W, Zhao W et al (2019) Joint prediction of breast cancer histological grade and Ki-67 expression level based on DCE-MRI and DWI radiomics. IEEE J Biomed Health Inform 24:1632–1642 31794406 10.1109/JBHI.2019.2956351

[26] Chen X, He L, Li Q et al (2023) Non-invasive prediction of microsatellite instability in colorectal cancer by a genetic algorithm-enhanced artificial neural network-based CT radiomics signature. Eur Radiol 33:11–22 35771245 10.1007/s00330-022-08954-6

[27] Su G-H, Xiao Y, You C et al (2023) Radiogenomic-based multiomic analysis reveals imaging intratumor heterogeneity phenotypes and therapeutic targets. Sci Adv 9:eadf0837 37801493 10.1126/sciadv.adf0837 PMC10558123

[28] Wu C, Zhang G, Wang L et al (2024) Spatial proteomic profiling elucidates immune determinants of neoadjuvant chemo-immunotherapy in esophageal squamous cell carcinoma. Oncogene 43:2751–2767 39122893 10.1038/s41388-024-03123-z

[29] Chiappa C, Greta M, Miriam L et al (2024) Neoadjuvant chemotherapy in breast cancer: evaluation of the impact on surgical outcomes and prognosis. Cancers (Basel) 16:2332 39001394 10.3390/cancers16132332 PMC11240326

[30] Shahabi F, Mehri A, Abdollahi A et al (2024) Post recurrence survival in early versus late period and its prognostic factors in rectal cancer patients. Sci Rep 14:17661 39085286 10.1038/s41598-024-67852-7 PMC11291732

[31] Kim S-Y, Cho N, Choi Y et al (2021) Factors affecting pathologic complete response following neoadjuvant chemotherapy in breast cancer: development and validation of a predictive nomogram. Radiology 299:290–300 33754824 10.1148/radiol.2021203871

[32] Pinard C, Debled M, Ben Rejeb H et al (2020) Residual cancer burden index and tumor-infiltrating lymphocyte subtypes in triple-negative breast cancer after neoadjuvant chemotherapy. Breast Cancer Res Treat 179:11–23 31529299 10.1007/s10549-019-05437-z

[33] Gu L, Liu Y, Guo X et al (2021) Computed tomography-based radiomic analysis for prediction of treatment response to salvage chemoradiotherapy for locoregional lymph node recurrence after curative esophagectomy. J Appl Clin Med Phys 22:71–79 34614265 10.1002/acm2.13434 PMC8598151

[34] Demirjian NL, Varghese BA, Cen SY et al (2022) CT-based radiomics stratification of tumor grade and TNM stage of clear cell renal cell carcinoma. Eur Radiol 1–12:32 10.1007/s00330-021-08344-4 34757449

