# Supplementary material for: Radiomic signatures associated with longitudinal TNM downstaging for prognostic stratification in breast cancer
Source: Insights Imaging. 2026 Apr 18;17:102. doi: 10.1186/s13244-026-02284-7 (PMC13091813; doi:10.1186/s13244-026-02284-7)
Supplement: Supplementary file 1 — ELECTRONIC SUPPLEMENTARY MATERIAL [file 13244_2026_2284_MOESM1_ESM.pdf]

Radiomic signatures associated with longitudinal TNM downstaging for prognostic stratification in breast cancer

ELECTRONIC SUPPLEMENTARY MATERIAL

Supplementary methods

Imaging protocol

For the prognostic development dataset, DCE-MRI data were obtained using SIEMENS 1.5T and GE 3T scanners, with all patients scanned in the prone position. The SIEMENS scanner acquired one precontrast image and five postcontrast images with a TR of 3.54–4.68 ms, a TE of 1.27–1.6 ms, a flip angle of 10° or 12°, an acquisition matrix of 448×448, and a slice thickness of 1.04–2.5 mm. The GE scanner acquired one precontrast image and four postcontrast images with a TR of 4.75–7.39 ms, a TE of 2.2–2.7 ms, a flip angle of 10°, an acquisition matrix of 512×512, and a slice thickness of 1.1–2.2 mm. Both scanners maintained a 120 s interval between postcontrast-enhanced phases.

In the prognostic validation 1 dataset, DCE-MRI data were obtained using Siemens 1.5T and GE 1.5T scanners with consistent imaging parameters: a minimum acquisition matrix of 256×192, 60 slices per series, a field of view of 16–18 cm, TR ≤ 20 ms, TE = 4.5 ms, flip angle ≤ 45°, slice thickness ≤ 3 mm, and pixel resolution ≤ 1 mm. Early enhancement series were acquired

approximately every 2 minutes postcontrast injection.

For the prognostic validation 2 dataset, DCE-MRI data were obtained using a 1.5T fat-suppressed MRI scanner (GE Healthcare, Milwaukee, Wisconsin, USA) with consistent imaging parameters: a minimum acquisition matrix of 256×192, 60 slices per series, a field of view of 18–20 cm, TR = 8 ms, TE = 4.2 ms, flip angle = 20°, slice thickness = 2 mm, and spatial resolution = 0.7×0.94×2.0 mm^3. Early enhancement series were acquired approximately every 5 minutes postcontrast injection.

Feature extraction

In this study, MRI feature extraction was performed using open-source PyRadiomics software [1]. A total of 107 radiomic features were systematically extracted from each image series, encompassing three distinct categories: (1) 18 first-order statistical features that describe the distribution of voxel intensities within the region of interest; (2) 14 morphological features that characterize the three-dimensional shape and geometric properties of the region; and (3) 75 texture features that quantify the spatial patterns and heterogeneity of intensity distributions.

Statistical Power Analysis

For training and evaluating the machine learning model, a moderate effect size with a statistical power of 80% and a significance level of α = 0.05 was used. The power analysis indicated that 99–175 samples would be sufficient for training and internal testing. Our study includes a development cohort (n=292)

for analysis, with 204 samples for model training and 88 samples for internal testing.

Additionally, post-hoc statistical power analysis was conducted to evaluate the model's generalizability and clinical applicability. For the ISPY1 dataset (n = 180), with an observed hazard ratio (HR) of 0.406 and an event rate of 26.1%, the statistical power was calculated to be 94.8%. Similarly, for the NACT-pilot dataset (n = 61), with an observed HR of 0.212 and an event rate of 37.7%, the statistical power was calculated to be 96.1%. These results demonstrate that the model maintains high statistical power across diverse datasets, further supporting its generalizability.

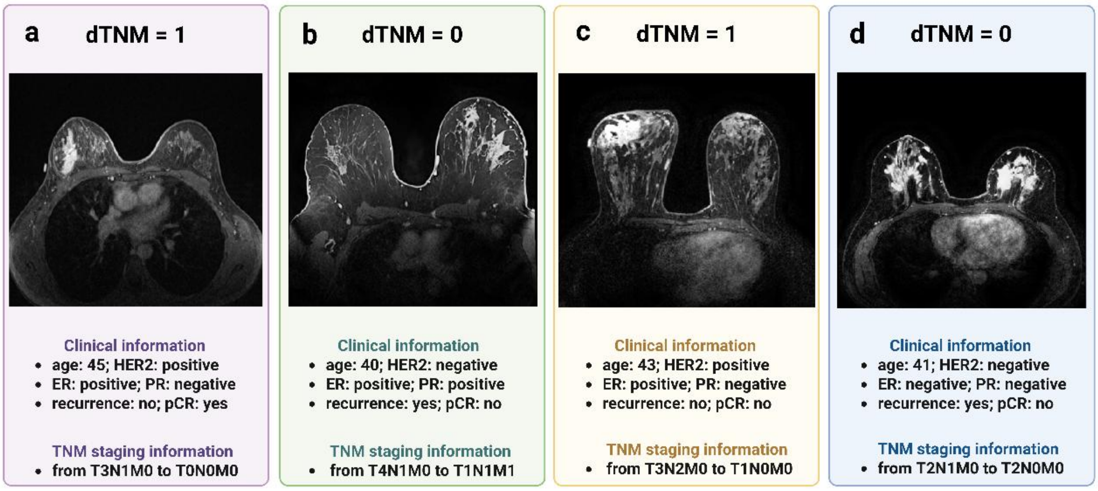

Supplementary Figure 1. Examples of Four Patients with TNM and clinicopathological Features

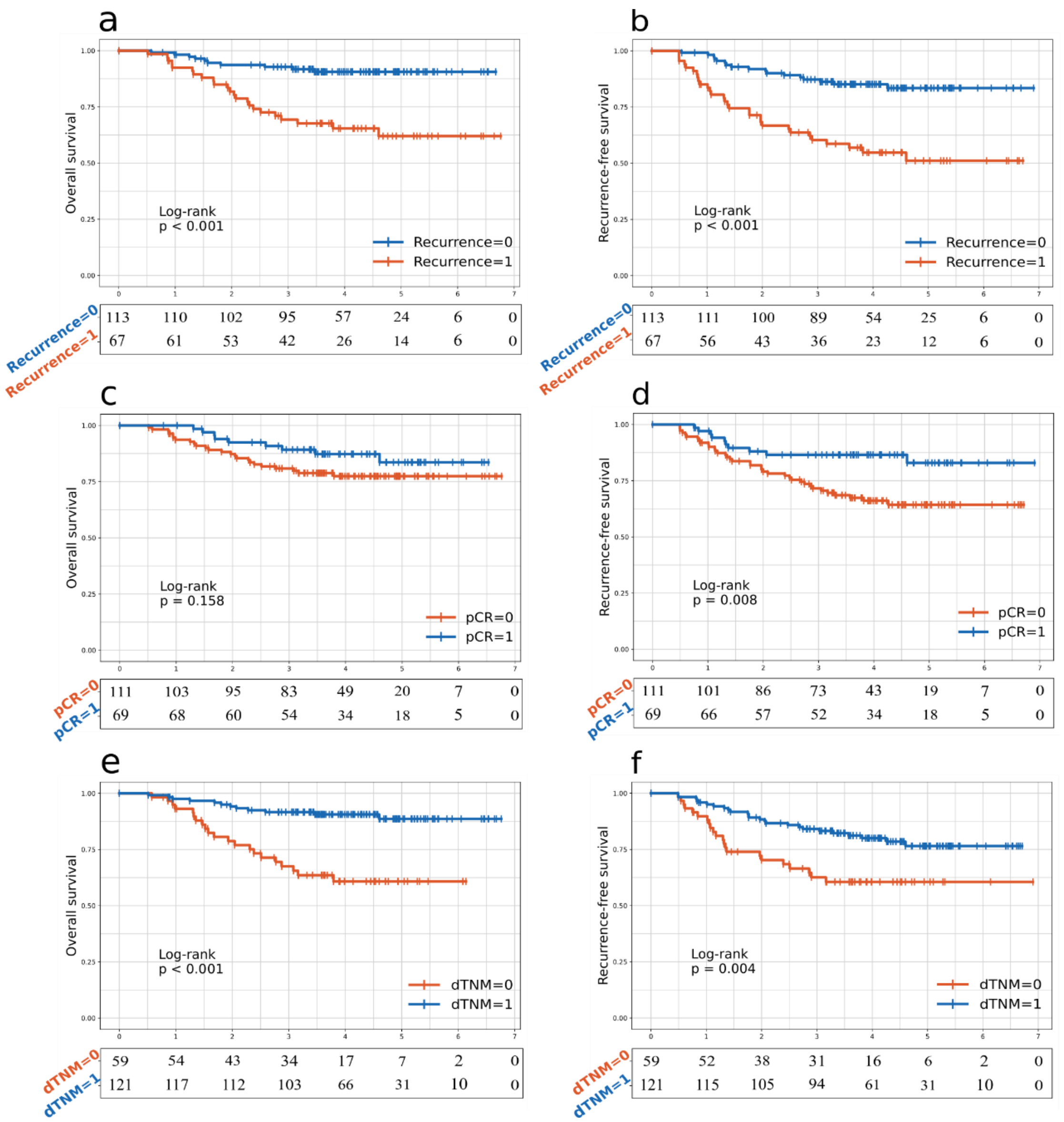

Supplementary Figure 2. External validation of imaging feature-predicted prognostic indicators based on prognostic validation 1 dataset: a) Recurrence vs. overall survival (OS) ($p < 0.001$); b) Recurrence vs. recurrence free survival (RFS) ($p < 0.001$); c) pCR vs. OS ($p = 0.158$); d) pCR vs. RFS ($p = 0.008$); e) dTNM vs. OS ($p < 0.001$); f) dTNM vs. RFS ($p = 0.004$); note: dTNM = 1 indicates TNM downstaging, whereas dTNM = 0 represents cases without downstaging.

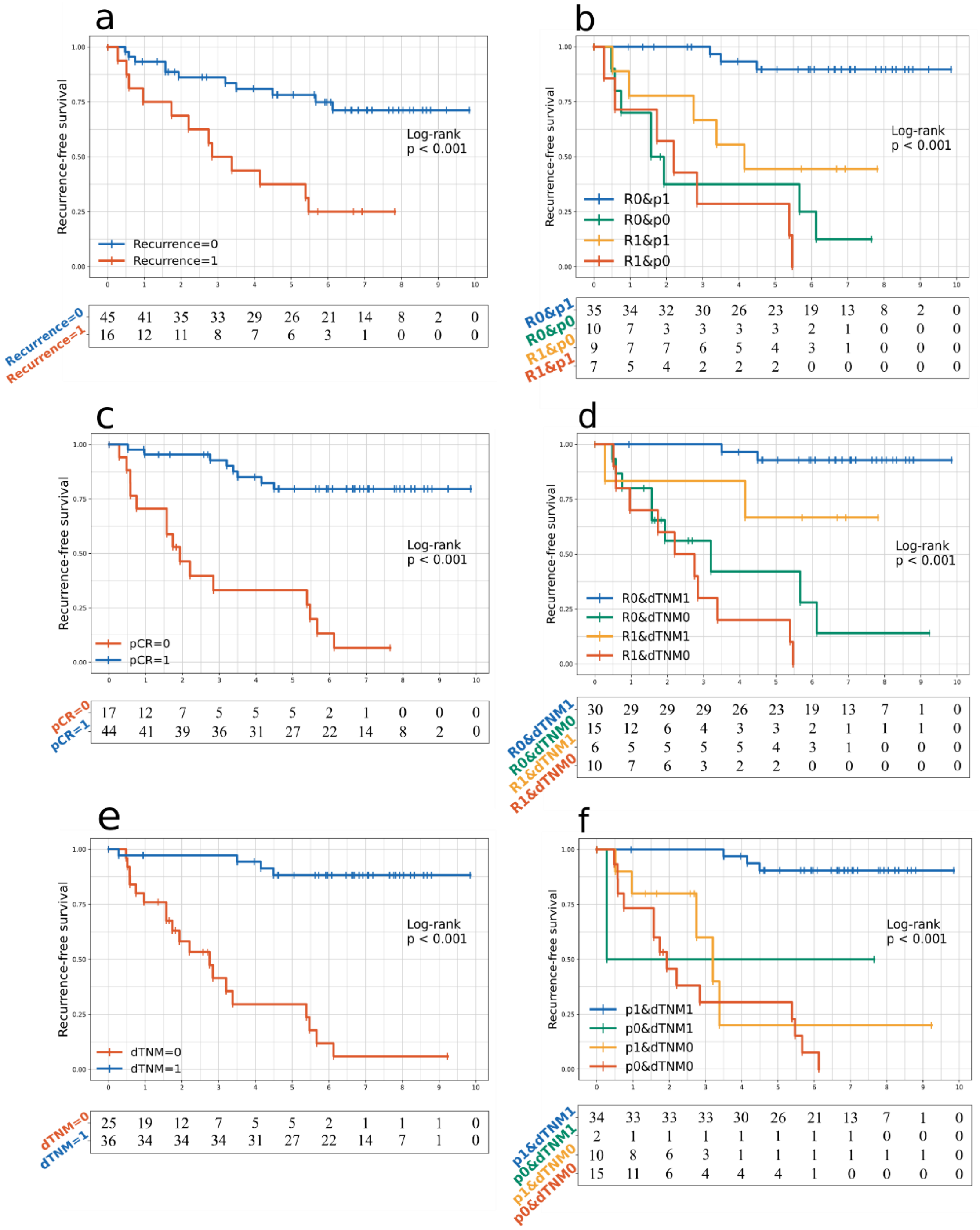

Supplementary Figure 3. External validation of imaging feature-predicted prognostic indicators based on the prognostic validation 2 dataset. Imaging-based model-predicted a) recurrence, c) pCR and e) dTNM predictions were utilized to evaluate recurrence free survival (RFS). The imaging-based model-predicted b) recurrence and pCR predictions were employed to assess RFS. Similarly, imaging-based model-predicted d) recurrence and dTNM predictions were applied to assess RFS. Additionally, imaging-based model-predicted f) pCR and dTNM predictions were utilized to evaluate RFS. R0 denotes recurrence = 0, R1 represents recurrence = 1; p0 indicates pCR = 0, p1 signifies pCR = 1;

dTNM0 corresponds to dTNM = 0, while dTNM1 stands for dTNM = 1.

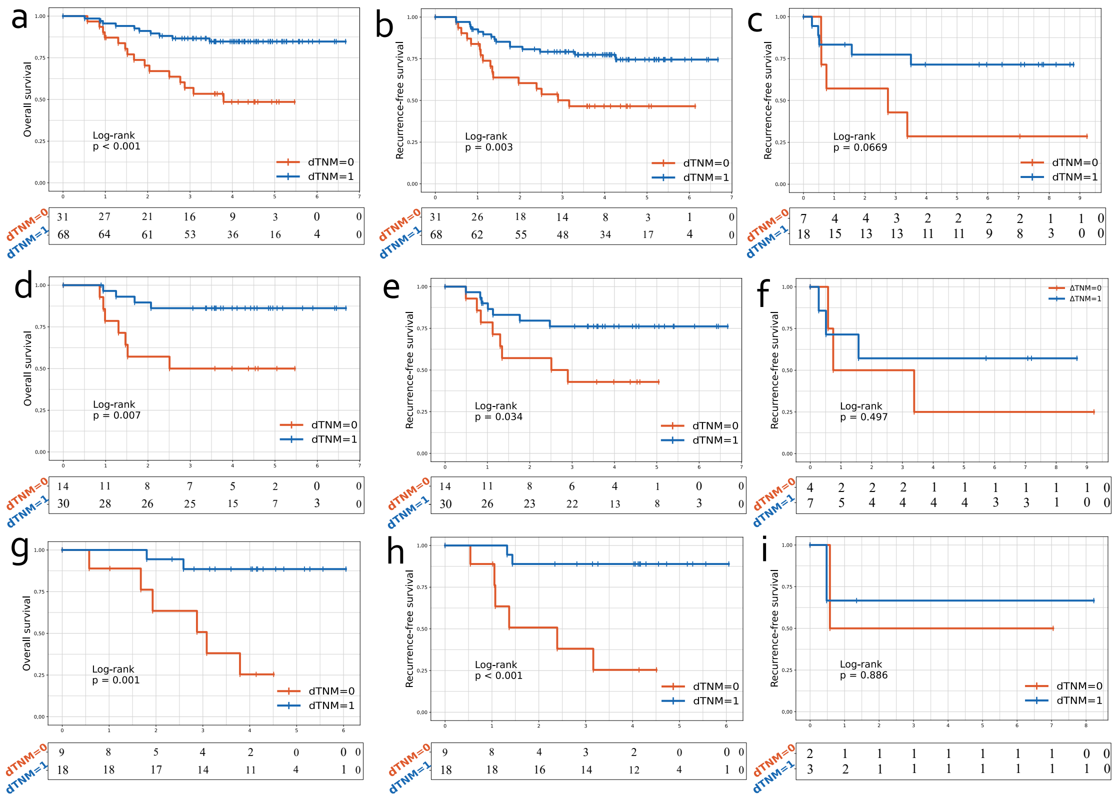

Supplementary Figure 4. External validation of imaging feature-predicted TNM downstaging (dTNM) indicators stratified by molecular subtype in prognostic validation 1 and prognostic validation 2 datasets. The hormone receptor (HR)-positive (ER and/or PR positive) subgroup: a) overall survival (OS) and b) recurrence-free survival (RFS) in the prognostic validation 1 dataset, and c) RFS in the prognostic validation 2 dataset. The triple-negative subgroup: d) OS and e) RFS in the prognostic validation 1 dataset, and f) RFS in the prognostic validation 2 dataset. The HER2-overexpression subgroup: g) OS and h) RFS in the prognostic validation 1 dataset, and i) RFS in the prognostic validation 2 dataset.

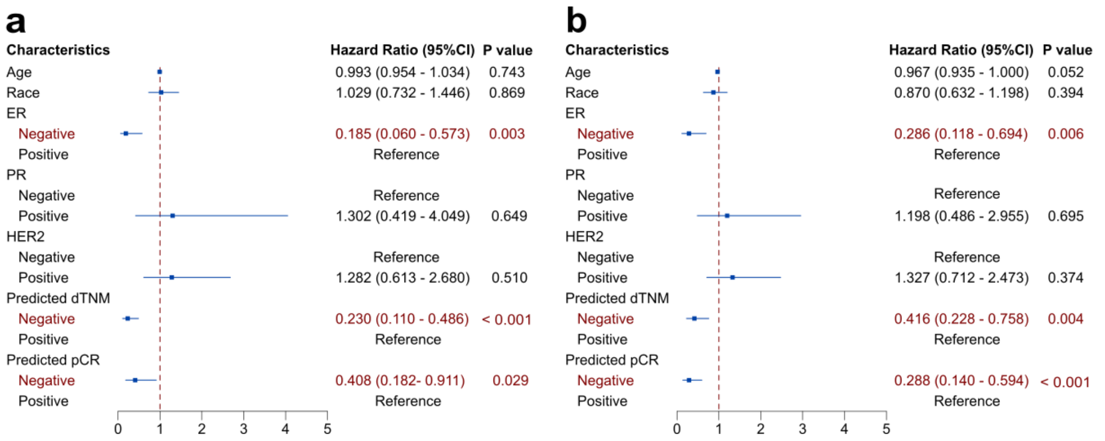

Supplementary Figure 5. Multivariate Cox Forest plot of prognostic indicators (dTNM and pCR), clinicopathological information in the prognostic validation 1 dataset showing associations with: a) overall survival (OS) and b) recurrence-free survival (RFS).

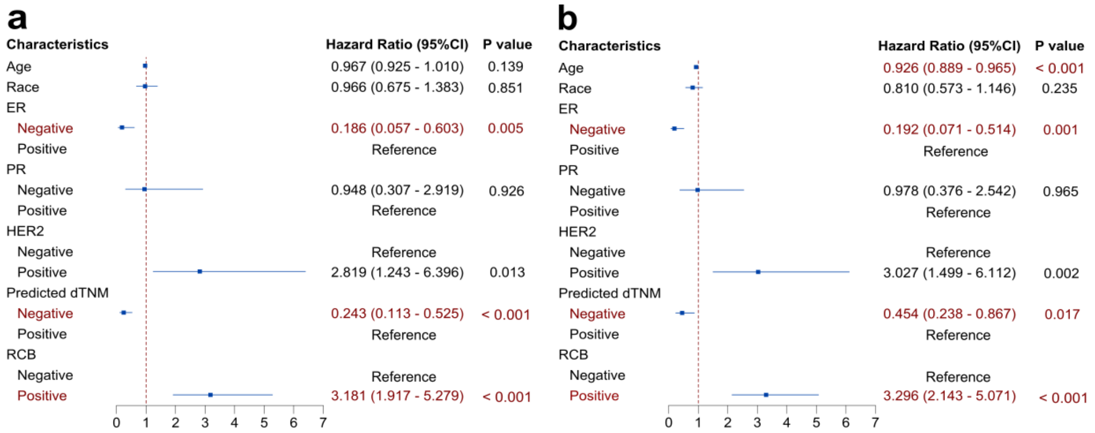

Supplementary Figure 6. Multivariate Cox Forest plot of dTNM, clinicopathological information, and residual cancer burden (RCB) in the prognostic validation 1 dataset showing associations with: a) overall survival (OS) and b) recurrence-free survival (RFS).

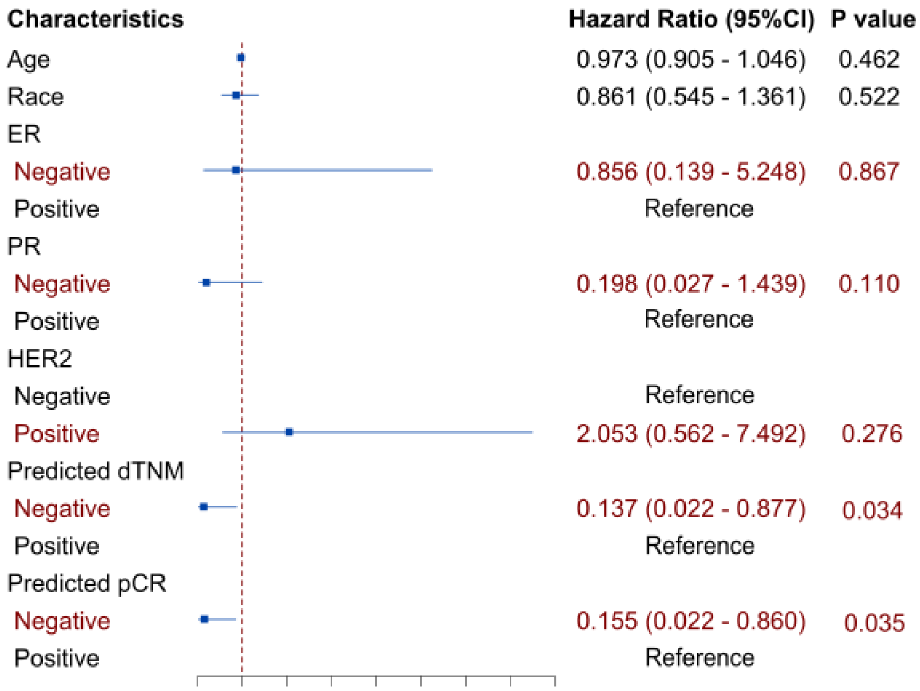

Supplementary Figure 7. Multivariate Cox Forest plot of prognostic indicators, clinicopathological information, and predicted pathological complete response in the prognostic validation 2 dataset showing associations with recurrence-free survival (RFS).

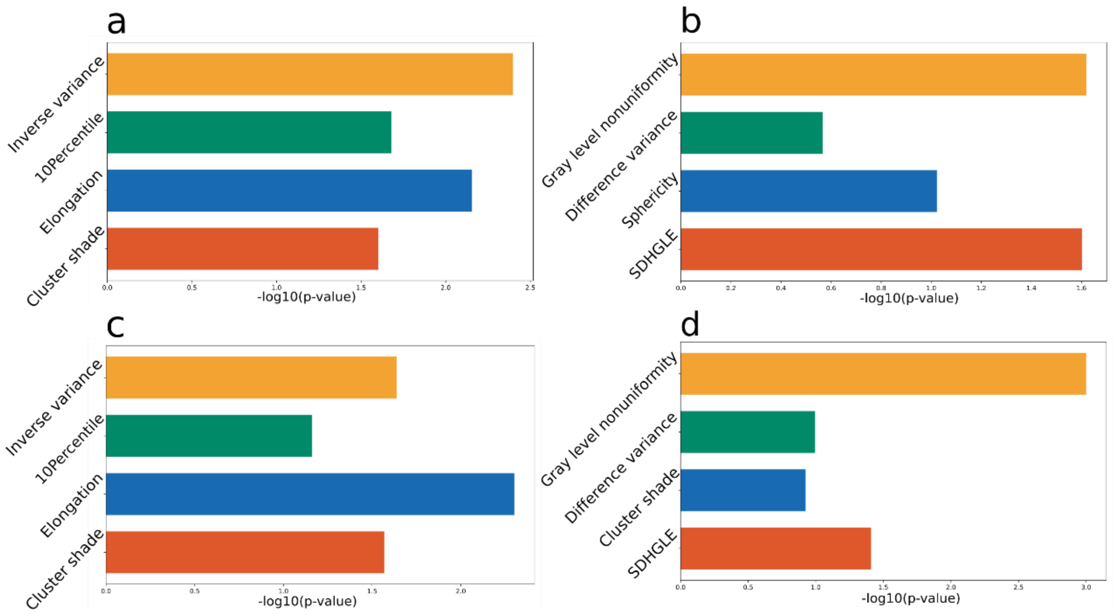

Supplementary Figure 8. Bar charts illustrating radiomic features associated with prognostic indicators in the prognostic validation 1 dataset. Radiomic features associated with pCR were correlated with a) overall survival (OS) and c) recurrence free survival (RFS) through survival analysis. The radiomic features associated with the dTNM were correlated with b) OS and d) RFS through survival analysis. The significance of the imaging features in survival is represented using -$\log_{10}$ (p value).

Supplementary Table 1. The integrated model for predicting prognosis indicators

	AUC (95% CI)	Accuracy (95% CI)	Specificity (95% CI)	Sensitivity (95% CI)
recurrence	0.916 (0.807 – 0.982)	0.909 (0.831 – 0.953)	0.911 (0.828– 0.956)	0.889 (0.565– 0.980)
pCR	0.806 (0.694 – 0.902)	0.784 (0.687 – 0.857)	0.790 (0.674 – 0.873)	0.769 (0.579 – 0.890)
dTNM	0.829 (0.725 – 0.922)	0.816 (0.714 – 0.887)	0.786 (0.605 – 0.898)	0.833 (0.704 – 0.913)

References

[1] J. J. Van Griethuysen, A. Fedorov, C. Parmar, A. Hosny, N. Aucoin, V. Narayan, R. G. Beets-Tan, J.-C. Fillion-Robin, S. Pieper, and H. J. J. C. r. Aerts, "Computational radiomics system to decode the radiographic phenotype," *Cancer Research,* vol. 77, no. 21, pp. e104-e107, 2017.
